# MUC1 Positive, Kras and Pten Driven Mouse Gynecologic Tumors Replicate Human Tumors and Vary in Survival and Nuclear Grade Based on Anatomical Location

**DOI:** 10.1371/journal.pone.0102409

**Published:** 2014-07-31

**Authors:** Tejas S. Tirodkar, Raluca A. Budiu, Esther Elishaev, Lixin Zhang, Jyothi T. Mony, Joan Brozick, Robert P. Edwards, Anda M. Vlad

**Affiliations:** 1 Department of Obstetrics, Gynecology and Reproductive Sciences, University of Pittsburgh School of Medicine, Pittsburgh, Pennsylvania, United States of America; 2 Magee Women's Research Institute, Pittsburgh, Pennsylvania, United States of America; 3 Department of Pathology, Magee-Women's Hospital, University of Pittsburgh Medical Center, Pittsburgh, Pennsylvania, United States of America; 4 Magee-Women's Hospital of the University of Pittsburgh Medical Center, Pittsburgh, Pennsylvania, United States of America; National Institutes of Health, United States of America

## Abstract

Activating mutations of Kras oncogene and deletions of Pten tumor suppressor gene play important roles in cancers of the female genital tract. We developed here new preclinical models for gynecologic cancers, using conditional (Cre-loxP) mice with floxed genetic alterations in Kras and Pten. The triple transgenic mice, briefly called MUC1KrasPten, express human MUC1 antigen as self and carry a silent oncogenic Kras^G12D^ and Pten deletion mutation. Injection of Cre-encoding adenovirus (AdCre) in the ovarian bursa, oviduct or uterus activates the floxed mutations and initiates ovarian, oviductal, and endometrial cancer, respectively. Anatomical site-specific Cre-loxP recombination throughout the genital tract of MUC1KrasPten mice leads to MUC1 positive genital tract tumors, and the development of these tumors is influenced by the anatomical environment. Endometrioid histology was consistently displayed in all tumors of the murine genital tract (ovaries, oviducts, and uterus). Tumors showed increased expression of MUC1 glycoprotein and triggered de novo antibodies in tumor bearing hosts, mimicking the immunobiology seen in patients. In contrast to the ovarian and endometrial tumors, oviductal tumors showed higher nuclear grade. Survival for oviduct tumors was significantly lower than for endometrial tumors (p = 0.0015), yet similar to survival for ovarian cancer. Oviducts seem to favor the development of high grade tumors, providing preclinical evidence in support of the postulated role of fallopian tubes as the originating site for high grade human ovarian tumors.

## Introduction

The American Cancer Society estimates over 91,000 new cases and 28,000 deaths due to gynecological cancers in 2013 [1]. Taken together, ovarian and endometrial tumors constitute about 78% of all female genital tract tumors. The most common gynecologic malignancy is endometrial cancer, which is often detected early and can be successfully treated with surgery and/or radiotherapy. In contrast, epithelial cancer of the ovary is relatively uncommon yet highly aggressive, accounting for most of the mortality. Primary fallopian tube cancers (without ovarian involvement) are also rare, accounting for 0.2% of cancer cases diagnosed annually [2] and, like ovarian tumors, are detected late and have a poor prognosis [3].

Traditionally, epithelial ovarian tumors have been thought to develop from the ovarian surface epithelium into four major histotypes: serous, endometrioid, mucinous and clear cell. It is now apparent that ovarian tumors are highly heterogeneous and may represent several different clinical entities, with distinct clinical precursors. High grade serous tumors carry p53 mutations and are considered to arise mostly in the fallopian tubes [4], [5]. Although this type of tumor has been fully characterized through The Cancer Genome Atlas (TCGA) [6], similarly comprehensive analyses of the other ovarian cancer subtypes are not yet available [7]–[9]. Nevertheless, based on substantial evidence from several studies, it is currently accepted that, at least in part, the endometrioid and clear cell ovarian tumor histotypes share endometriosis as a putative common precursor [10] and display frequent inactivating mutations in ARID1A [7], [11].

Low grade (type I) endometrial and ovarian cancers, as well as tubal intraepithelial carcinomas are frequently associated with oncogenic KRAS^G12D^ and PTEN deletion mutations [3], [12] or altered expression [13]. The recent TCGA study of 373 endometrial tumors identified the KRAS and PTEN genes as being mutated in 24.6% and 77% of endometrioid tumors respectively, emphasizing the influence of these mutations in gynecologic cancer pathogenesis [14].

Involvement of the KRAS and PTEN pathways has led to the development of several genetically modified preclinical models for type I gynecological cancers [15]–[20]. Using conditionally transgenic mice carrying both oncogenic Kras^G12D^ and a floxed Pten deletion, Dinulescu et al demonstrated the importance of these two pathways in triggering ovarian tumors with endometrioid histology [21]. Mice defective in Pten have also been reported as valuable preclinical models for endometrioid endometrial tumors [22]. However, in vivo modeling of oviduct tumors (the murine equivalent of fallopian tube tumors) has proven more challenging with only few orthotopic models reported to date [15], [23].

While some of the mouse models for gynecologic malignancies have been helpful in delineating mechanisms of pathogenesis [21], [24], [25], they offer limited utility for immunotherapy due to the absence of well characterized mouse tumor antigens. To overcome this, we generated triple transgenic MUC1^+/−^loxP-STOP-loxP-Kras^G12D/+^Pten^loxP/loxP^ (or briefly MUC1KrasPten) mice that, at steady state, express physiologic levels of human mucin 1 (MUC1) as self-antigen [26]. MUC1 is a membrane-bound glycoprotein that is overexpressed and aberrantly glycosylated in most epithelial cell-derived cancers, including genital tract tumors [27]. MUC1-targeted immunotherapy is under development for several cancers and has been administered so far to about 1200 patients, while more than 2000 patients are currently enrolled in ongoing clinical trials [28]. Using the MUC1KrasPten mouse model, we have recently demonstrated that intrabursal injections of AdCre (to activate oncogenic Kras and induce Pten loss in the ovaries) [21], [26] trigger endometrioid ovarian tumors. The tumors overexpress human MUC1 similarly to the human disease and respond to MUC1 immunotherapy, further strengthening the evidence on its efficacy as a target in ovarian cancer [26].

Here, we show how conditional mutations in Kras and Pten genes can be manipulated throughout the genital tract of double (KrasPten) and triple transgenic (MUC1KrasPten) mice, using injections of Cre-encoding adenovirus (AdCre) in the ovarian bursa, oviduct or uterine horns. Although all tumors, regardless of the originating site, display endometrioid histology, oviducts seem to favor the development of high grade tumors, providing preclinical evidence in support of the postulated role of fallopian tubes as the originating site for high grade, human ovarian tumors.

## Materials and Methods

### Survival surgery and administration of recombinant adenovirus for tumor induction

**Figure 1:**
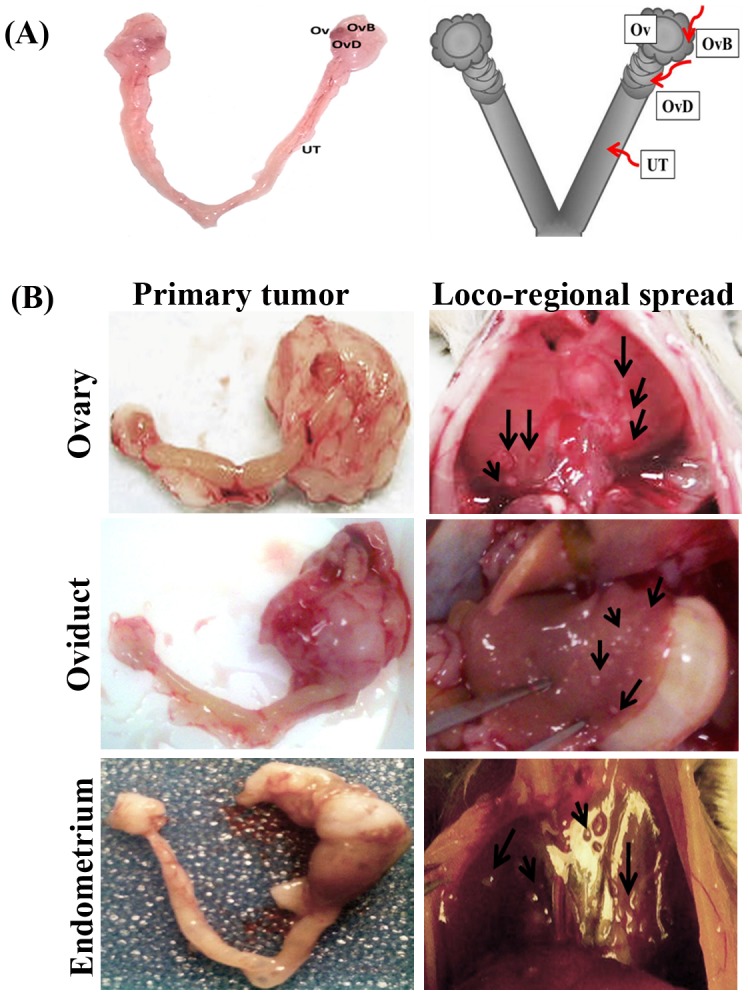
Induction of floxed Kras^G12D^ and Pten mutations in the female genital tract results in large tumors at the primary site, accompanied at late stages by numerous peritoneal implants. (A) Left panel: female genital tract anatomy of a healthy mouse (Ov, ovaries; OvB, ovarian bursa; OvD, oviduct; UT, uterus). Right panel: schematic representation of the murine female genital tract showing the ovaries, oviducts and uterine horns. Arrows indicate AdCre delivery routes: under the ovarian bursa, inside the oviduct and inside the uterine horn. All injections were unilateral, keeping the contralateral site as control. (B) Mice carrying conditional mutations in oncogenic Kras^G12D^ and tumor suppressor Pten pathways were injected with AdCre either under the left ovarian bursa (n = 12), left oviduct (n = 9), or left uterine horn (n = 12). Representative gross images of primary tumors (left column) are shown for ovarian, oviductal and uterine injections. Right column shows numerous loco- regional metastatic small tumor deposits (arrows) that accompany the corresponding primary tumor. The nodules were often located on the diaphragm (upper and lower panels) and liver (middle panel).

All animal experiments were performed according to the protocol approved by the University of Pittsburgh Institutional Animal Care and Use Committee. Figure 1 shows the gross anatomy of the murine female genital tract from a healthy mouse, as well as a diagram of the ovarian bursa, oviduct and uterine sites of AdCre injection approach.

Briefly, 7–10 weeks old female mice were synchronized by intra-peritoneal (IP) injection of 5 U of pregnant mare serum gonadotropin (PMSG, Sigma, St. Louis, MO), followed 48 hours later by 5 U of human chorionic gonadotropin (hCG, Calbiochem, Billerica, MA) as previously described [21]. Thirty six hours later, 5 µl of 2.5×10^7^ plaque-forming units of Ad5CMVCre (University of Iowa Gene Transfer Vector Core) were delivered into either the ovarian bursa of the left ovary (n = 12), or the left oviduct (n = 9) or the left uterine horn (n = 12). The contra lateral (right) ovary/oviduct/uterine horn was used as control. A subset of oviduct injections (n = 4) were performed after clipping the oviduct at the proximal and distal ends using the GEM MicroClips (Synovis Life Technologies, Birmingham, AL) to ensure retention of the adenovirus within the oviduct.

The mice were sacrificed when the tumor mass on the injected side and/or ascites accumulation became visible or the mice showed signs of distress that were pre-defined as endpoints (i.e. hunched appearance, ruffled fur, difficulty in reaching for food or water etc).

### Administration of AdLacZ adenovirus and staining for LacZ expression

Ovulation was synchronized as above in control female mice. Five microliters of 2.5×10^7^ plaque-forming units of the AdLacZ adenovirus (University of Iowa Gene Transfer Vector Core) were then delivered into proximally and distally clipped oviducts (n = 2) or the uterus (n = 2). Mice were sacrificed 3–7 days post virus administration and the tissues fixed and stained for LacZ expression using the LacZ Detection Kit for Tissues, according to the manufacturer's instructions (Invivogen, San Diego, CA). After staining, the tissues were embedded in paraffin and blocks were sectioned at 5 µm, followed by H&E staining to visualize the histology of the AdLacZ infected sites.

### Tissue isolation, histopathology and immunohistochemistry

Mouse internal organs (reproductive tract, spleen, peritoneal tumor masses and diaphragm), blood and ascites were collected during necropsy. Harvested tissue was fixed in 10% buffered formalin (Fisher Scientific, Kalamazoo, MI) for 24 hours, stored in 70% ethanol for 3 days and subsequently embedded in paraffin. Five micron sections were cut and the gross histopathology was assessed by H&E staining. For immunohistochemistry (IHC), the slides were blocked using 3% hydrogen peroxide in methanol and antigen retrieval was performed by boiling the slides for 20 minutes in citrate buffer, pH 6. The following antibodies were used for IHC: anti-human MUC-1 (HMPV, 1∶100, BD Pharmingen, San Diego, CA), cytokeratin 8 (B0017, 1∶50, Assay Biotech, Sunnyvale, CA), and desmin (sc7559, 1∶50, Santa Cruz Biotechnology, Dallas, TX). Secondary antibodies used include anti-rabbit-HRP (K4003, Dako, Carpinteria, CA) for cytokeratin 8, and anti-goat-HRP for desmin (sc2020, 1∶50, Santa Cruz Biotechnology, Dallas, TX). Biotinylated anti-mouse IgG (550337, 1∶100; BD Pharmingen, San Jose, CA) was used as the secondary antibody for anti-MUC1, followed by the VectaStain ABC Kit (Vector Laboratories, Burlingame, CA). The positive signal was detected using the DAB chromogen (DAB Substrate Kit, Abcam, Cambridge, MA) and the slides were counterstained using hematoxylin. To ensure specificity of staining, control sections were stained with either isotype control antibodies or no primary antibody.

Human serous tubal intraepithelial carcinoma (n = 1), human endometrioid endometrial carcinoma (n = 4) and human endometrial hyperplasia (n = 3) were obtained as per IRB guidelines from the Health Science Tissue Bank of the Magee Women's Hospital, Pittsburgh. The protocols for processing and IHC staining of human tumors were similar to those described above, for mouse tumors.

### Image processing and analysis

Images were acquired with the Nikon Eclipse 90i microscope and Nikon DS-Ri1 CCD camera, using NIS Elements AR software or the Nikon Eclipse 600 microscope with the DS-L3 CCD camera. Images acquired were processed with Adobe Photoshop CS5.

### DNA isolation and PCR analysis of Cre-mediated recombination

DNA was isolated from 5 µm tissue sections of primary tumors using All Prep RNA/DNA/Protein isolation kit as per the manufacturer's instructions (Qiagen, Valencia, CA). Tails from non-tumor bearing, healthy control mice were snap-frozen after collection and DNA was later isolated using Puregene DNA purification system (Gentra Systems, Minneapolis, MN), according to manufacturer's instructions. The primers and complete PCR protocols to detect K-ras^G12D^ and Pten deletion mutations have been described previously [26].

### Flow cytometry

Spleens were collected at necropsy and a single cell suspension was obtained by passing the tissue fragments through a 70 µm cell strainer (BD Falcon, Franklin Lake, NJ, USA). Cells were stained with fluorescent antibodies for CD3 (PerCP), CD4 (Pacific Blue), and CD8 (APC-Cy7) (all antibodies from BD Biosciences, San Jose, CA), followed by intracellular staining for Foxp3 (eBioscience, San Diego, CA), according to the manufacturers' protocols.

To detect anti-MUC1 antibodies, samples were incubated with IG10-MUC1 cells [29] expressing extracellular human MUC1. To detect bound antibodies, the cells were then stained with fluorescein tagged anti-mouse IgG and positive cells analyzed with LSRII (BD Biosciences) and processed in FACSDiva (BD Biosciences). Gates for positive cells were set using control ascites, from tumor bearing KrasPten (i.e. human MUC1 negative) mice.

### ELISA

To detect MUC1-specific antibodies in sera from MUC1KrasPten mice with tumors (n = 5 ovarian, n = 4 oviductal and n = 4 uterine tumors) we performed ELISA, as previously described by us and others [29]. Briefly, ELISA plates were coated with 10 µg/ml 100mer MUC1 peptide comprising five 20 amino-acid long tandem repeats from the MUC1 extracellular domain. Similarly diluted sera from two mice with MUC1 negative (wild type) tumors, as well as dilution medium alone were chosen as negative controls. Samples were run in duplicate for each of the two dilutions (1∶20 and 1∶40, respectively). Horseradish peroxidase (HRP) –conjugated secondary antibody specific for mouse IgG (Sigma, 1∶500) was used for detection. Median and standard errors were plotted in Excel.

### Survival curve and statistical analysis

The Kaplan-Meyer survival curve was plotted using the GraphPad Prism 6 software. The same software, as well as Excel were used to perform ANOVA or Student's t test and to compute p values for statistical significance.

## Results

### Induction of oncogenic Kras^G12D^ and deletion of Pten in the oviduct or the uterine horns triggers progression to ductal and endometrial tumors, respectively

To explore the tumorigenic contributions of oncogenic Kras and tumor suppressor Pten pathways throughout the female genital tract of genetically engineered, Cre-loxP mice [21], [26], we injected AdCre adenovirus at three different anatomical locations (Fig. 1A). The mice received one, unilateral AdCre injection either in the ovarian bursa (n = 12), oviduct (the fallopian tube equivalent, n = 9) or uterine horn (n = 12). Activation of Kras and deletion of Pten transformed the oviductal and endometrial tissues, resulting in establishment of primary tumors at these sites (Fig. 1B). Oviductal tumors showed 100% tumor penetrance (n = 9), similarly to ovarian tumors [21], [26]. Tumor penetrance was lower (at 50%) following intrauterine (IU) AdCre injections. Intrabursal injections triggered primary ovarian tumors as shown previously [21], [26] and were used as reference standard (Fig. 1B).

Gross loco-regional metastases were often observed in late stage tumors of oviduct and uterus (Fig. 1B) and were detected as tumor implants on the diaphragm, liver, and spleen. Only one of the 12 uterine-injected mice presented with ascites (8%) while 5 out of 9 oviduct injected mice showed ascites (56%, p = 0.0163). Ascites, when detected, was of the hemorrhagic type. No tumors were detected in the ovaries, oviduct and uterine-AdCre injected mice carrying mutations in either Kras alone (MK mice) or Pten alone (MP mice, data not shown), suggesting that, as with ovarian tumors [26], both pathways need to be active in order for tumors to occur. AdCre was also injected in MUC1 single transgenic mice (included as controls) which, as expected, remained healthy throughout the duration of the experiment. This demonstrates that in the absence of floxed mutations in the host genome, adenoviral infection is non-consequential for the host.

DNA of all primary tumors was analyzed by PCR to confirm the activation of the floxed sites in Kras and Pten genes [26]. As expected, the oviductal and uterine tumors showed the presence of both the active Kras^G12D^ and the wild type Kras alleles, along with homozygous deletion of Pten (Fig. S1A–C). Normalization results of mutant (floxed) Kras to wild type Kras from tumors are in line with the expected 1∶1 ratio (50% mutant Kras), although the oviduct and endometrial show slightly lower and higher ratios, respectively (Fig. S1D). A similar efficiency of recombination was observed for floxed Pten that could be detected in these lesions (Fig. S1E). In contrast, no wild type was detected, consistent with the fact that macro-dissected tissue consisted mostly of epithelium. However, a weak band for wild type Pten could be detected in the ex vivo isolated ovarian tumor cell line using freshly isolated DNA, also suggesting the negative result can in part be due to a limitation in detecting residual Pten by PCR when using paraffin-extracted DNA (Fig. S1F). Through reporter gene (AdLacZ) experiments, we further confirmed that injections remain anatomically confined and effectively trigger local epithelial infection of uterus, oviduct and ovaries (Fig. S2).

### Oviductal and uterine tumors are of epithelial origin and show endometrioid histology

**Figure 2:**
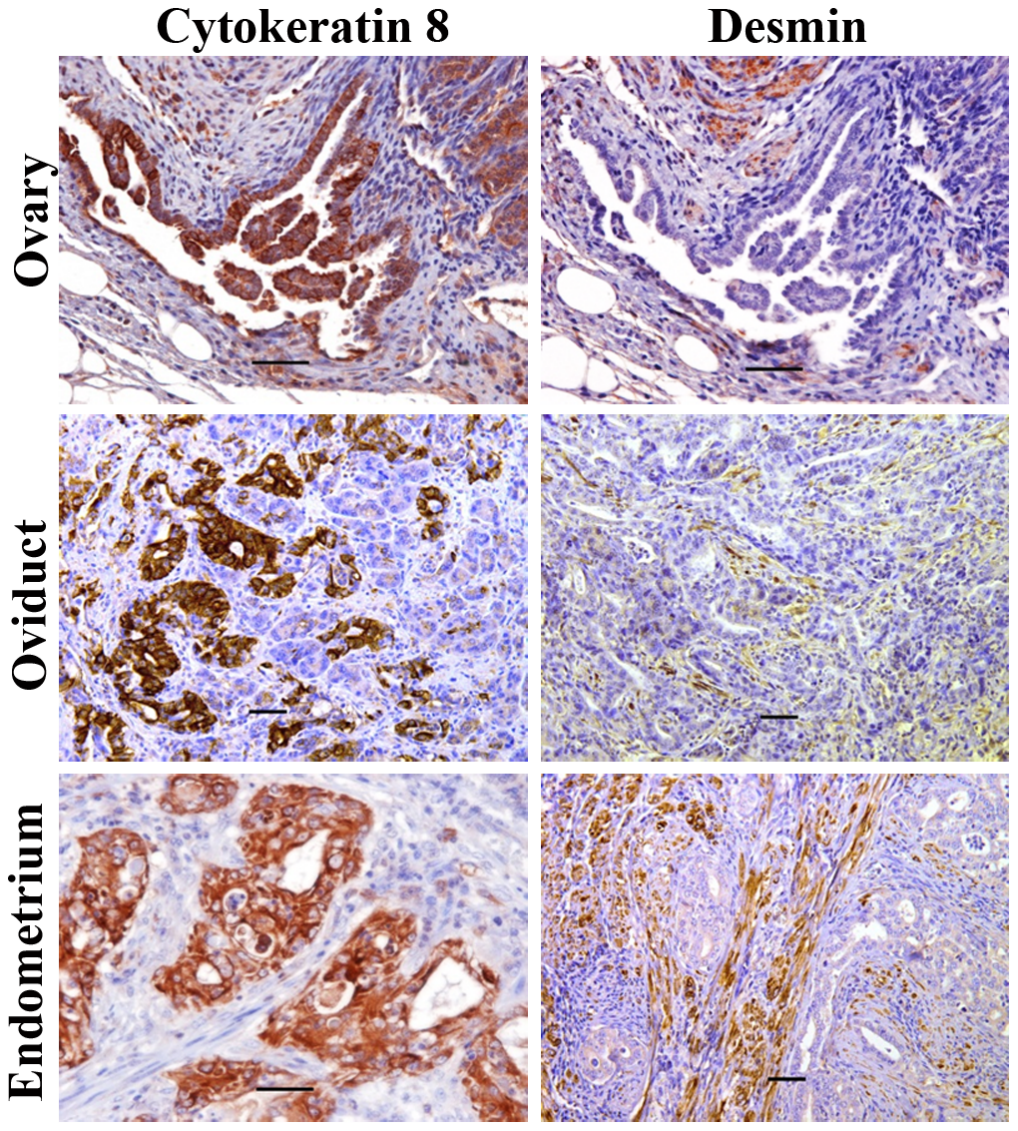
Primary tumors of the ovary, oviduct and uterus have epithelial origin. Immunohistochemistry staining of tumors occurring in the ovary (upper panels), oviduct (middle panels) or endometrium (lower panels). Antibodies to mouse cytokeratin 8 (an epithelial cell marker, left column) and mouse desmin (right column) were used at 1∶50 dilution. Representative images shown. Scale bar −50 µm.

**Figure 3:**
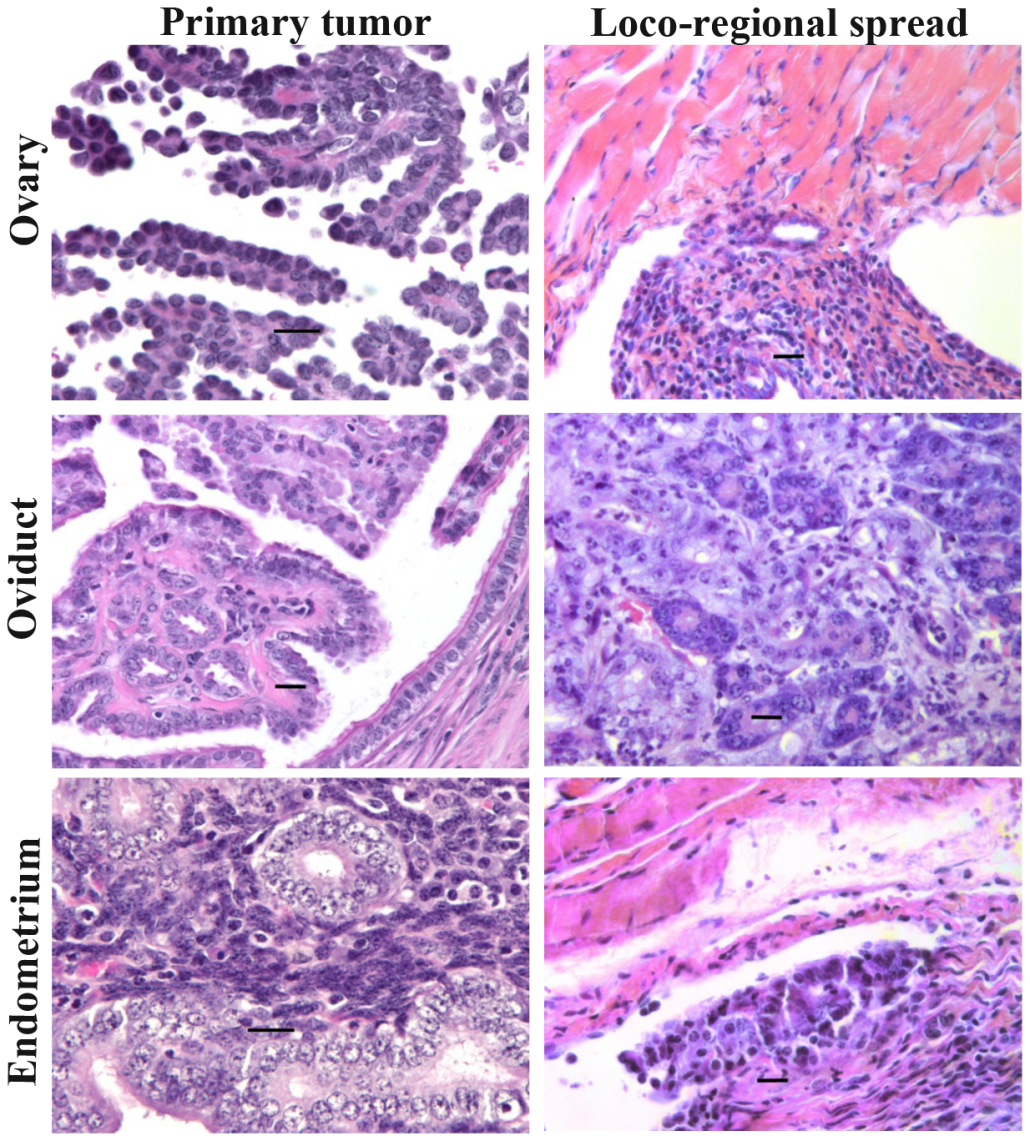
Oviductal and endometrial tumors show endometrioid histology at both primary and satellite locations. Formalin fixed and paraffin embedded primary and metastatic tumor tissues were analyzed for histo-pathology. Representative images of H&E stained tumor sections are shown. Left column: primary tumors of the genital tract show endometrioid histology in the ovary, oviduct and endometrium. Right column: secondary tumors, including ovarian metastases to the diaphragm (upper), oviduct metastases to the pancreas (middle) and endometrial metastases to the diaphragm (lower) also show endometrioid histology. Scale bar −20 µm.

Both oviductal and uterine tumor cells were positive for cytokeratin 8, an epithelial marker, and negative for desmin, a stromal cell marker (Fig. 2), confirming the epithelial origin of these tumors. Similarly to KrasPten- induced ovarian tumors obtained via AdCre injections under the ovarian bursa [21], both the oviductal and uterine primary tumors displayed endometrioid histology (Fig. 3). The endometrioid histology was also preserved in loco-regionally spread tumor implants (Fig. 3). Our findings demonstrate that, in this preclinical model, co-involvement of Kras and Pten tumorigenic pathways throughout the genital tract (ovaries, oviduct and uterus) consistently triggers gynecologic tumors with endometrioid histology. Notably however, some of the uterine lesions in mice sacrificed early, potentially before tumor onset, showed glandular hyperplasia with cystic dilation (Fig. S3).

### KrasPten-driven oviductal and uterine tumors express human MUC1 and trigger spontaneous anti-MUC1 antibodies

**Figure 4:**
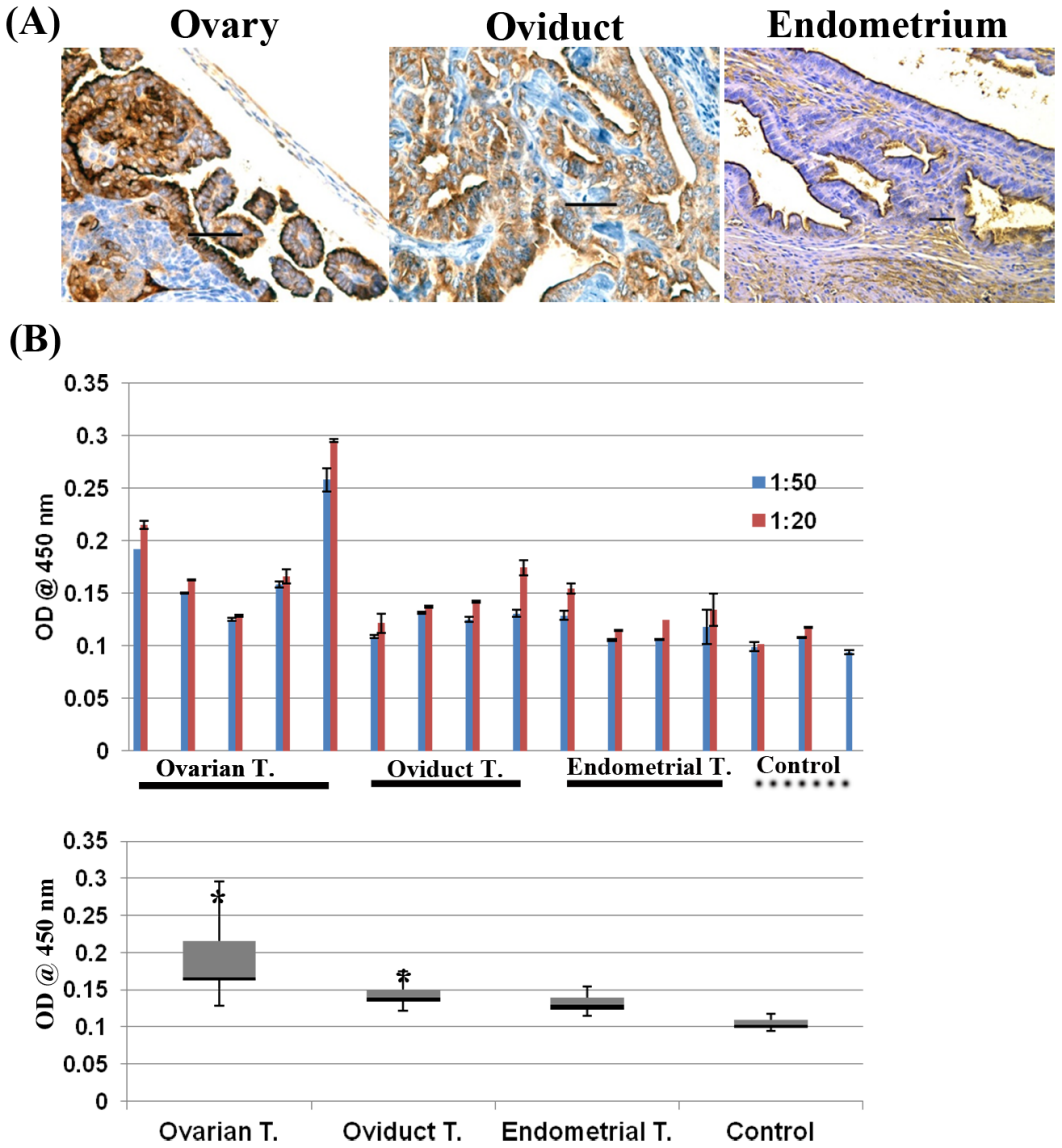
Increased human MUC1 protein expression in Kras- and Pten- driven genital tract tumors of MUC1KrasPten triple transgenic mice triggers humoral immunity. (A) MUC1 immunohistochemistry staining of tumors occurring in the ovary (upper panel), oviduct (middle panel) or endometrium (lower panel). An antibody specific to the human MUC1 extracellular domain (clone HMPV, mouse IgG1) was used at 1∶100. Polarized MUC1 expression throughout the genital tract of healthy female mice at baseline is shown in Fig. S2. Mouse tumor MUC1 mimics human tumor expression (shown in Fig. S5). Representative immunohistochemical images shown. Scale bar −50 µm. (B) ELISA measurement of human MUC1 peptide-specific IgG antibodies in sera from MUC1KrasPten mice with tumors (n = 5 ovarian, n = 4 oviductal and n = 4 uterine). Upper panel: presence of antibodies at two different dilutions, using sing as target peptide a 100mer peptide comprising fie 20-aminoacid long peptide from the MUC1 extracellular domain of MUC1. Background levels were detected using sera from KrasPten mice with MUC1 negative tumors (i.e. wild -type for MUC1). Vehicle only was also included as an additional negative control. The assay was run in duplicate and values were plotted as means with standard deviations. Lower panel: box and whisker diagrams (min, Q1, median, Q3, max) of readings at 1∶20 dilution. Antibody levels are significantly higher (compared to control readings) in the ovarian and oviduct tumor group (one way ANOVA p<0.05; *two tail t test; p<0.05). Uterine tumors, p = 0.052.

We have previously shown that triple transgenic MUC1KrasPten mice, injected with AdCre under the ovarian bursa, develop human MUC1-expressing ovarian tumors, closely mirroring the human disease [21], [26]. In this study, we examined whether the oviductal and uterine tumors also expressed MUC1 upon Kras activation and Pten deletion in MUC1KrasPten mice. Our IHC results demonstrate that the tumors lost polarized MUC1 expression normally seen on healthy epithelia (Fig. S4), and show abundant cell surface and cytosolic MUC1 (Fig. 4), similar to the staining pattern observed in human tumors (Fig. S5 and references [30], [31]).

In patients, MUC1 overexpression on developing adenocarcinomas leads to spontaneous humoral responses to various MUC1 epitopes from its extracellular domain [32], [33]. We asked here whether the gynecologic tumors in MUC1KrasPten mice that express MUC1 antigen as self also trigger MUC1-specific humoral immunity. ELISA measurements show that although the amplitude of the response varies, presence of MUC1-specific IgG antibodies can be detected in serum of tumor bearing mice (Fig. 4B) and the levels are significantly higher in mice with ovarian and oviduct tumors. Since the target peptide is a 100mer MUC1 peptide comprising five tandem repeats from the extracellular portion of MUC1, these responses are indicative of humoral immunity against underglycosylated, tumor-like MUC1, as previously shown by us and others [26]. Furthermore, mice with endometrial hyperplasia also have detectable levels of MUC1-specific antibodies, suggesting that MUC1 humoral immunogenicity is an early event, triggered by early precursors (Fig. S3D).

Overall, these results demonstrate that MUC1KrasPten mice represent the first immune competent, orthotopic, human MUC1-expressing preclinical tumor model for epithelial cell- derived oviduct and endometrial tumors. The tumors have well defined (endometrioid) histology, and, as with ovarian tumors [26], overexpress MUC1 and trigger detectable levels of spontaneous MUC1-specific humoral responses, closely mirroring the immunogenicity seen in the respective human diseases [32], [33].

### KrasPten- induced oviductal and uterine tumors differ in their nuclear grade, survival and immune microenvironment

**Figure 5:**
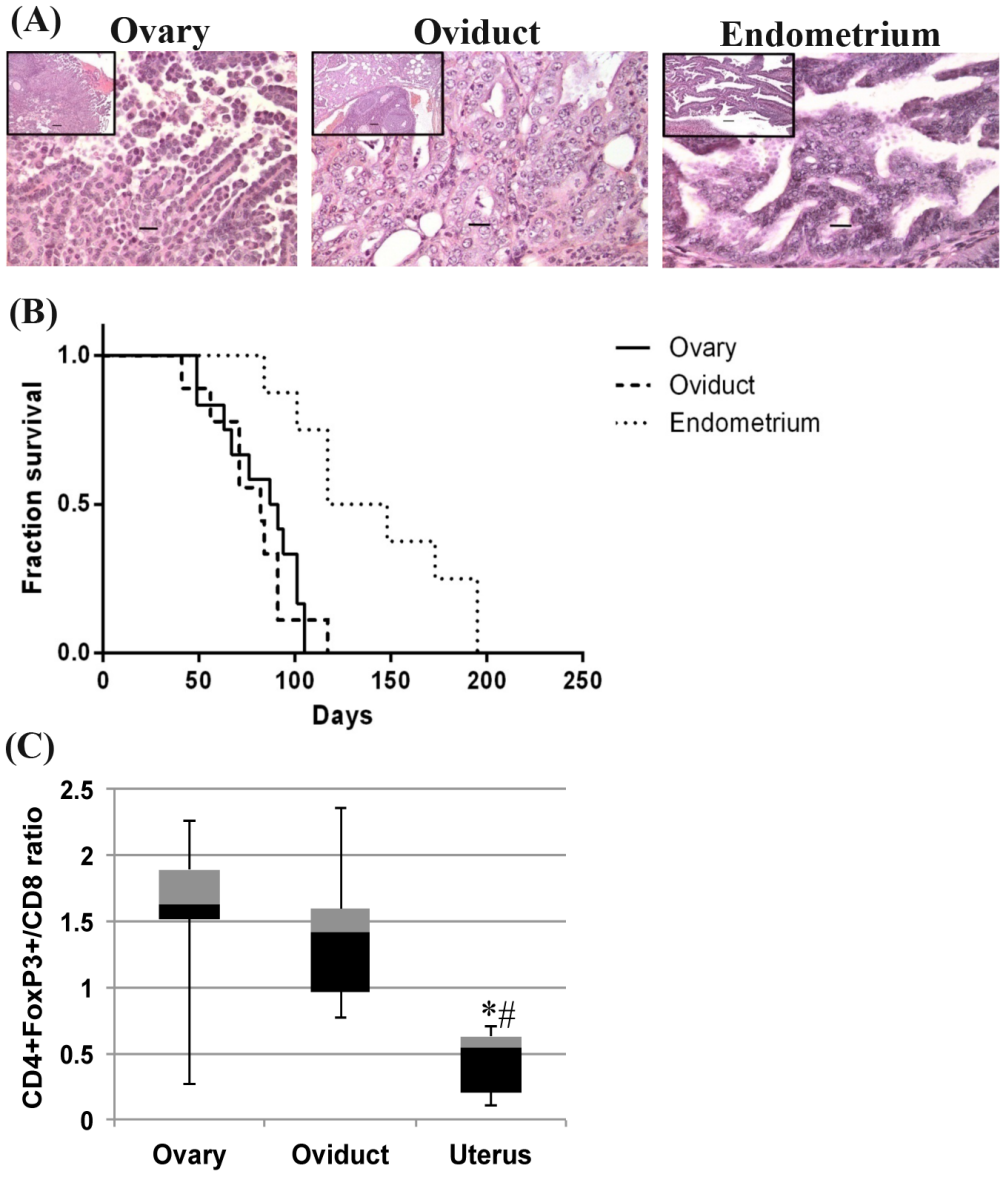
Kras- and Pten- induced tumors differ in nuclear grade and survival based on the anatomical site of mutation activation. (A) Nuclear grade of primary tumor tissues of the ovary, oviduct and the uterus. Representative H&E images are shown. Scale bars: Main −20 µm, Inset −100 µm. (B) Kaplan Meyer curve shows that mice with uterine tumors survive significantly more than those with ovarian tumors (* p = 0.0015) or those with ductal tumors (# p = 0.0016). Individual group comparison after post ANOVA Bonferroni correction (p<0.016). The numbers of mice in each tumor group and median survival time for each tumor type are listed Table S1. Mice with premalignant lesions in the uterine tumor category were excluded from analyses. (C) Splenic Treg/CD8 T cell ratios in mice with ovarian, oviduct or uterine tumors (n = 5 mice/group), represented as box and whisker diagrams (min, Q1, median, Q3, max). CD4 and CD8 T lymphocytes were gated under the CD3 population. Foxp3 cells were gated under the CD4 population. One way ANOVA for comparison of all means (p<0.03) and two tail t tests between any two groups show significant differences between the ratios in uterine tumors and any of the other two tumor types, ovarian and oviduct (p<0.02 and p<0.01, respectively).

Although all genital tract tumors were endometrioid, a detailed analysis of the H&E histo-pathology revealed that only oviduct tumors developed as poorly differentiated, high nuclear grade tumors, in contrast to the uterine and ovarian tumors which occurred primarily as low/intermediate grade tumors (Fig. 5A). Furthermore, mice with oviduct tumors had the lowest median survival (12 weeks), significantly shorter than mice with endometrial tumors (Fig. 5B, p = 0.001). Surprisingly, no significance was reached when compared with mice bearing ovarian tumors (13 weeks median survival, Table S1). Thus, the ovarian and oviductal tumors mirror the characteristics of the human ovarian [1] and fallopian tube cancers and share a similarly low survival, in spite of the high nuclear grade observed only in the latter.

To explore the relationship between survival, the observed phenotype of oviduct tumors and the immune status of the host, we phenotyped the splenic T cells via multicolor flow cytometry and analyzed the percentages of all CD8 and CD4 T lymphocytes, and of Foxp3 + (Treg) subset. The ratio of suppressors (Tregs) to effectors (CD8 T lymphocytes) has been shown to correlate inversely with survival of patients with ovarian [34] or other types of tumors [35]–[37]. In line with these reports, we observed an increased Foxp3 + T cell accumulation in the spleen of oviduct tumor- bearing mice and a higher ratio of Treg/CD8 in these mice compared to mice with uterine tumors (Fig. 5C, p<0.01). No differences were noted between mice with ovarian and oviduct tumors, suggesting that both anatomical locations are similar in inducing an immune suppressive phenotype in the host, despite the high nuclear grade observed only in the latter.

## Discussion

Studies on targeted therapies, including immune-based approaches, require the development of adequate preclinical models that best reflect the pathogenic changes seen in the human disease. In this study, we generated two novel human MUC1- expressing mouse models of oviductal and endometrial cancers respectively, based on simultaneous Kras^G12D^ activation and Pten deletion mutations [21], [26]. Using triple transgenic MUC1KrasPten mice [26] we show for the first time that concomitant activation of oncogenic Kras and deletion of Pten tumor suppressor throughout the female mouse genital tract consistently triggers MUC1 positive epithelial tumors with endometrioid histology. We previously showed that MUC1 distribution throughout the genital tract of MUC1KrasPten healthy mice is similar to the one seen in women [26]. Here, we demonstrate that progression to genital cancers triggers loss of polarized distribution and significant increase in MUC1 protein expression. Furthermore, these changes trigger humoral immune responses, most likely due to the release of MUC1 from tumor cell surface followed by expansion of MUC1-specific B cells in tumor draining lymph nodes [32], [33], [38]. Patients with premalignant and malignant conditions of the genital tract (mostly uterus and ovaries), as well as those affected with other cancers [39] have increased MUC1 antibody titers, although the intensity of these responses is variable. In line with these findings, the triple transgenic MUC1KrasPten mice employed here, which express human MUC1 as self, are able to undergo similar pathogenic changes leading to local (ascites) and systemic (serum) IgG antibody responses in ovarian, tubal and endometrial tumors. This demonstrates the versatility of MUC1KrasPten mice in modeling, with high fidelity, immunobiology of MUC1 in gynecologic cancers.

A second major finding of our studies stems from the fact that although the same genetic changes were turned on, at a similar rate, throughout the genital tract epithelium (in the ovaries, oviduct, endometrium), the tumor microenvironment seems to be a key determinant of tumor grade and survival. Though contiguous with the uterus and the ovary, and triggered via the same Kras^G12D^ and Pten^del^ mutations, the oviductal tumors show a higher nuclear grade than those arising in the other organs. In women, the high grade serous ovarian tumors are believed to arise from fallopian tubes [4], [5], [15], [40]. This hypothesis is further validated by recent preclinical studies from Perets et al. who reported a genetic model of de novo high grade serous carcinoma that originates in fallopian tube epithelium and recapitulates the biology of human invasive ovarian cancer [23]. Our results raise the previously unexplored possibility that fallopian tubes may also play a causative role in (albeit rare) cases of high grade endometrioid or mixed endometrioid-serous ovarian carcinomas. There are several examples in human carcinogenesis where the anatomical site of initiating lesions dictates the cancer risk, including the cervical epithelial transformation zone with HPV [41], the esophageal-gastric junction and Barrett's esophagus [42], and the squamous cell metaplasia in lung cancer [43]. Here we report that the intrinsic nature of the mucoso-epithelial biology of the fallopian tube may promote a more aggressive phenotype, as compared to the adjacent uterine mucosa when exposed to the same carcinogenic influence. Our approach opens the door for future studies focused on the identification of fallopian tube-specific molecular pathways engaged in tumorigenesis and development of new therapies that target these pathways. It also provides further support to the rationale of scrutinizing the fallopian tubes when searching for premalignant or early precursors to high grade ovarian tumors, regardless of their histology.

Preclinical mouse models to study oviductal cancer, the murine equivalent of human fallopian tube carcinoma, are difficult to develop. Recent studies from Kim et al utilized the anti-müllerian hormone receptor 2 (Amhr2) gene locus to deliver the Cre recombinase and conditionally delete the Dicer and the Pten genes in the müllerian ducts to establish ductal cancer closely resembling the human disease [15]. However, the Amhr2 gene is expressed not only in oviductal cells but also in the uterine epithelium as well as the ovarian granulosa cells [19], making this model non-exclusive for primary oviductal tumors. Similarly, we acknowledge the technical challenges posed by induction of oviduct tumors in our MUC1KrasPten mice. Oviducts are minute tubes that provide a space continuum between the uterine horns and the ovarian bursa. To diminish the risk of leakiness, and to ensure that AdCre injections remain anatomically confined to the oviducts, we clipped the proximal and distal ends of the tubes, prior to AdCre injections. The contrasting histomorphology (high grade in oviducts versus low grade in ovaries and uterus) suggests that the originating cells were indeed from the oviduct and that tumors were not merely spreading from the contiguous genital tract areas (ovary and uterus, respectively).

Unlike oviduct tumors, several preclinical models of uterine carcinomatosis are currently available. Conditional deletions of tumor suppressors such as Pten and p53 in the endometrium trigger invasive endometrial adenocarcinomas [17], [18]. In line with these studies, our endometrial cancer mouse model did not show 100% penetrance. Mice with no visible tumors showed signs of endometrial hyperplasia. The lesions were immunogenic and triggered MUC1 antibodies, making this model attractive for studies on MUC1 in uterine premalignancy.

The tumor microenvironment, composed of stroma and immune cells, has recently received emphasis as a target in treatment of ovarian cancer [44]. Our study reinforces its role in the development of gynecological cancers. In addition, oviduct tumor-bearing mice have Treg to CD8 ratios that are higher than in mice with uterine tumors, yet not significantly different from mice with ovarian tumors. This suggests that the oviducts promote a more immune suppressive environment, perhaps similarly to ovaries, via CXCL12 [45], although the exact mechanisms remain to be identified.

Taken together, our studies establish two new, highly versatile human MUC1- expressing mouse models of Kras- and Pten- induced oviductal and endometrial cancers with endometrioid histology, which closely mirror the pathology and immunogenicity of human disease, and demonstrate the influence of the tumor microenvironment on gynecological cancer development.

## Supporting Information

Figure S1 Cre-mediated recombination at Kras and Pten loci, in tumor-extracted DNA. PCR analysis of tumor-extracted DNA shows concomitant activation of oncogenic KrasG12D mutation (A) and deletion of Pten (B). Non-deleted Pten is shown in (C). DNA from a healthy transgenic mouse was used as negative controls in and B and positive control in C. DNA from an ovarian cancer cell line was used as positive control in A and B. (A) Floxed out, activated Kras shows up as upper band. (B) Floxed out Pten shows as a single band; no band demonstrates absence of Cre-loxP recombination. (C) Wild type Pten allele (arrow). (D) Activated Kras levels expressed as percentage of total K-Ras in each sample. (E, F) Pten deletion and wild type Pten allele, respectively; y axis, signal intensity (arbitrary units). Signal in D-F were quantified using Image Studio Lite (LI-COR). Ov T; ovarian tumor; Od T; oviduct tumor; Endom; endometrial tumor.
(TIF)

Figure S2 AdLacZ administration into the oviduct or the uterus was performed followed by staining for β-galactosidase expression. 4 micron sections of the specific tissue were cut and HE stained to reveal the tissue histology. β-galactosidase expression in epithelia of oviduct and the endometrium indicate successful delivery of the adenovirus. A representative section is shown for each oviduct and uterine anatomical site (Scale bars: low magnification −100 µm, high magnification −50 µm.
(TIF)

Figure S3 (A) Baseline endometrial histology of a healthy mouse. (HE stain) (B) Premalignant lesions display cystic dilation and endometrioid hyperplasia. (HE) stain) (C) The cyst lining as well as the hyperplasic endometrial glands express human MUC1 (IHC for MUC1 using anti-human MUC1 antibody, clone HMPV). Scale bar −200 µm. (D) Dot plot of IG10-MUC1 cells incubated with serum from uterine injected female mouse with endometrial hyperplasia. Gated population represents percent tumor cells stained by MUC1-specific antibodies present in the serum.
(TIF)

Figure S4 Histomorphology and MUC1 expression in the normal mouse female genital tract. Left column: HE stain of a female genital tract of a healthy, MKP mouse showing normal, baseline histology of the ovary, oviduct and the uterus. Right column: IHC stain for human MUC1 expression in the ovary, oviduct and uterus of a healthy MKP female mouse. Scale bar −50 µm.
(TIF)

Figure S5 Histomorphology and MUC1 expression in human gynecologic tumors. Left column: HE stains of human fallopian tube carcinoma, endometrial carcinoma and endometrial hyperplasia. Right column: IHC stain for human MUC1 expression. Representative images shown. Scale bar −50 µm.
(TIF)

Table S1 Median survival and number of mice in each tumor group.
(DOCX)
