# Supplementary figures and images for: MUC1 Positive, Kras and Pten Driven Mouse Gynecologic Tumors Replicate Human Tumors and Vary in Survival and Nuclear Grade Based on Anatomical Location
Source: PLoS One. 2014 Jul 31;9(7):e102409. doi: 10.1371/journal.pone.0102409 (PMC4117479; doi:10.1371/journal.pone.0102409)

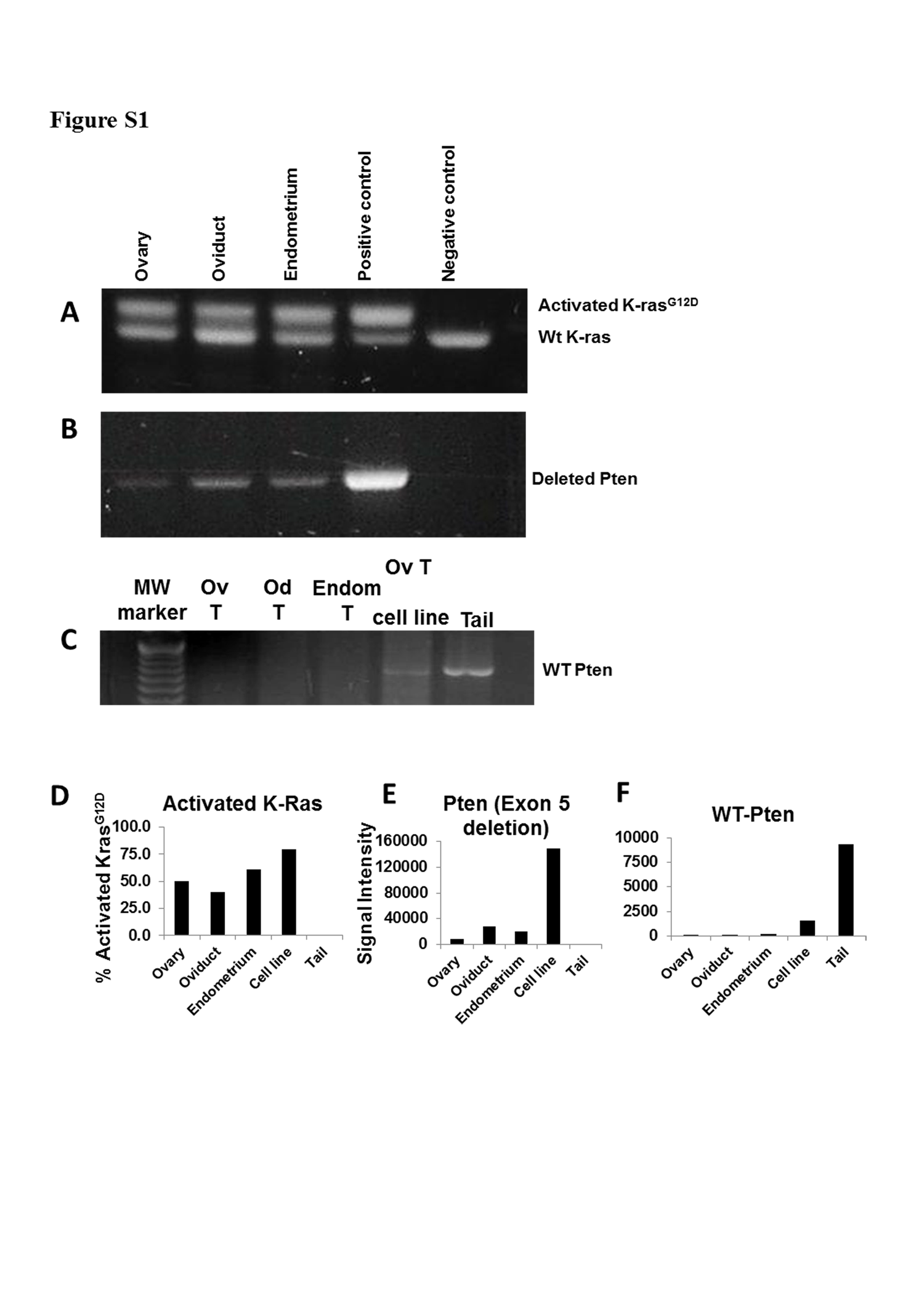

Supplement: Figure S1 — Cre-mediated recombination at Kras and Pten loci, in tumor-extracted DNA. PCR analysis of tumor-extracted DNA shows concomitant activation of oncogenic KrasG12D mutation (A) and deletion of Pten (B). Non-deleted Pten is shown in (C). DNA from a healthy transgenic mouse was used as negative controls in and B and positive control in C. DNA from an ovarian cancer cell line was used as positive control in A and B. (A) Floxed out, activated Kras shows up as upper band. (B) Floxed out Pten shows as a single band; no band demonstrates absence of Cre-loxP recombination. (C) Wild type Pten allele (arrow). (D) Activated Kras levels expressed as percentage of total K-Ras in each sample. (E, F) Pten deletion and wild type Pten allele, respectively; y axis, signal intensity (arbitrary units). Signal in D-F were quantified using Image Studio Lite (LI-COR). Ov T; ovarian tumor; Od T; oviduct tumor; Endom; endometrial tumor. (TIF) [file pone.0102409.s001.tif]

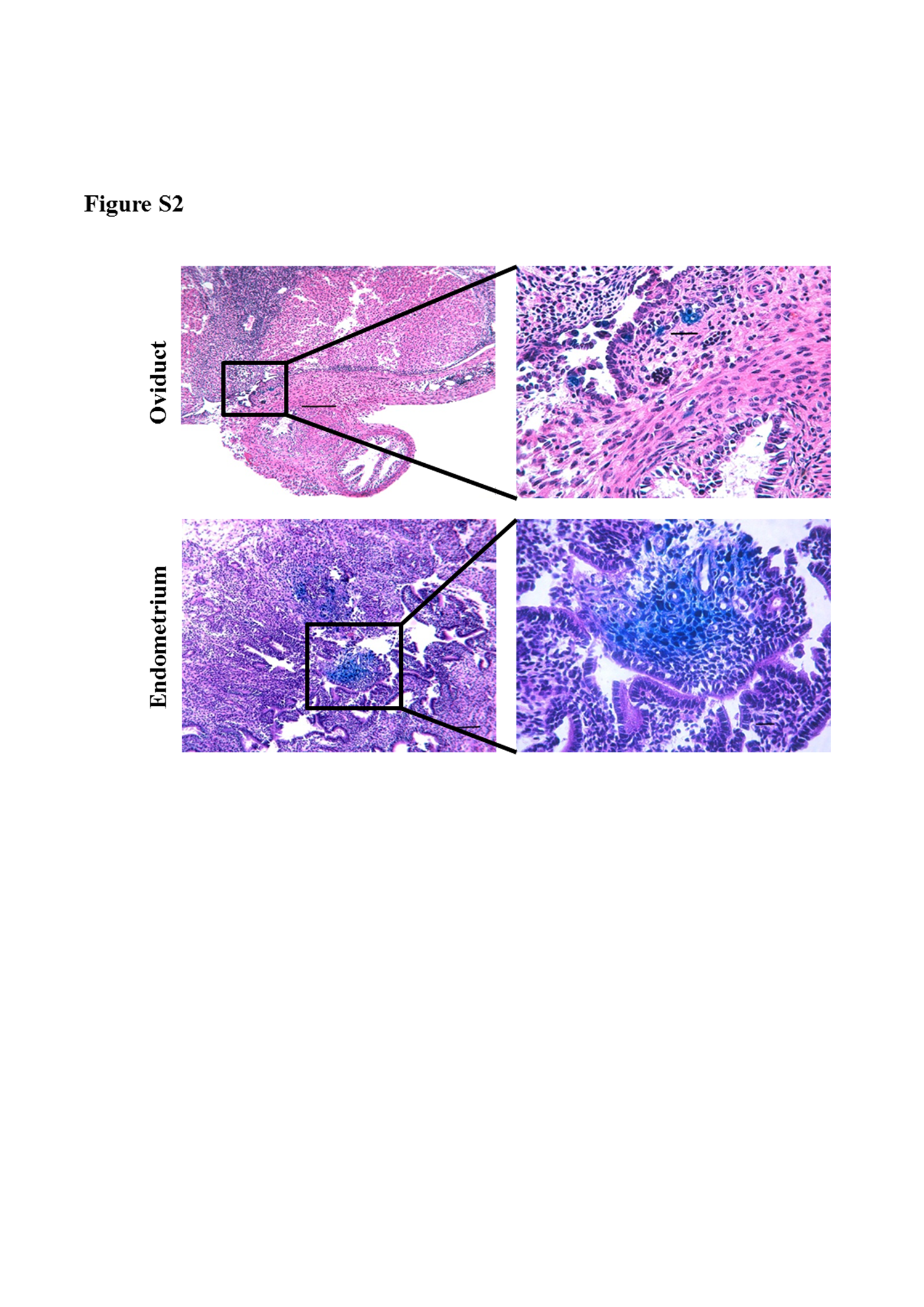

Supplement: Figure S2 — AdLacZ administration into the oviduct or the uterus was performed followed by staining for β-galactosidase expression. 4 micron sections of the specific tissue were cut and HE stained to reveal the tissue histology. β-galactosidase expression in epithelia of oviduct and the endometrium indicate successful delivery of the adenovirus. A representative section is shown for each oviduct and uterine anatomical site (Scale bars: low magnification −100 µm, high magnification −50 µm. (TIF) [file pone.0102409.s002.tif]

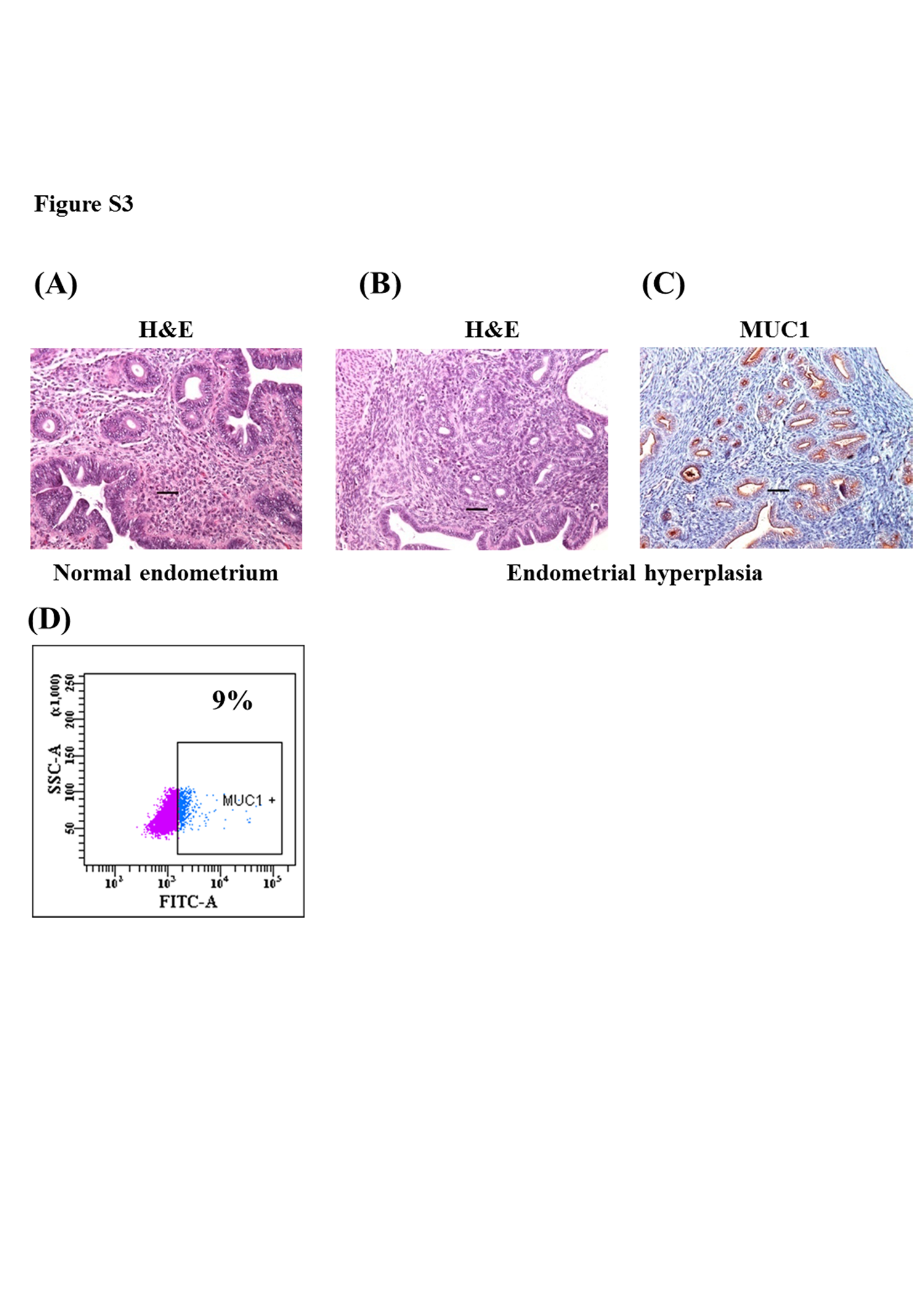

Supplement: Figure S3 — (A) Baseline endometrial histology of a healthy mouse. (HE stain) (B) Premalignant lesions display cystic dilation and endometrioid hyperplasia. (HE) stain) (C) The cyst lining as well as the hyperplasic endometrial glands express human MUC1 (IHC for MUC1 using anti-human MUC1 antibody, clone HMPV). Scale bar −200 µm. (D) Dot plot of IG10-MUC1 cells incubated with serum from uterine injected female mouse with endometrial hyperplasia. Gated population represents percent tumor cells stained by MUC1-specific antibodies present in the serum. (TIF) [file pone.0102409.s003.tif]

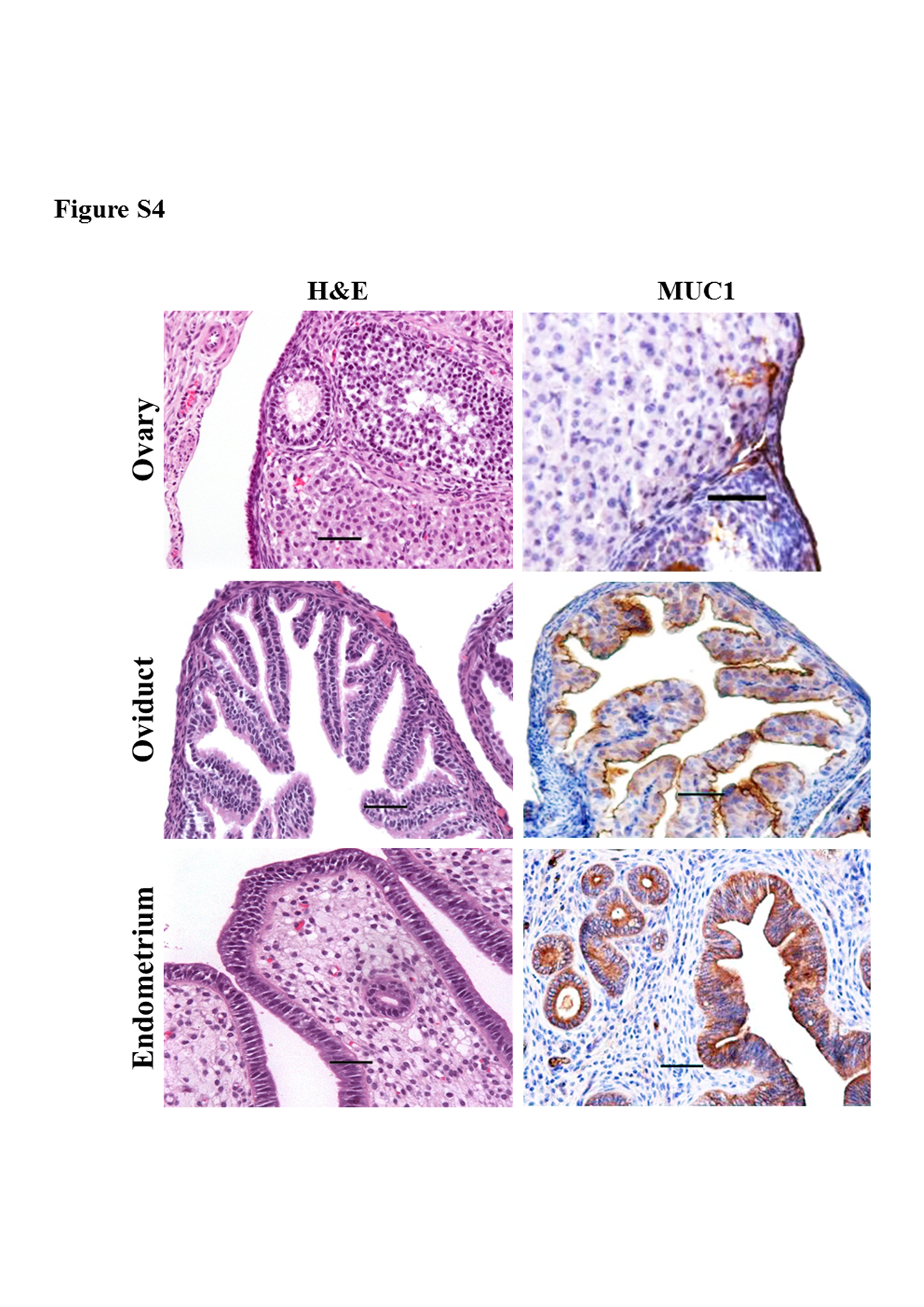

Supplement: Figure S4 — Histomorphology and MUC1 expression in the normal mouse female genital tract. Left column: HE stain of a female genital tract of a healthy, MKP mouse showing normal, baseline histology of the ovary, oviduct and the uterus. Right column: IHC stain for human MUC1 expression in the ovary, oviduct and uterus of a healthy MKP female mouse. Scale bar −50 µm. (TIF) [file pone.0102409.s004.tif]

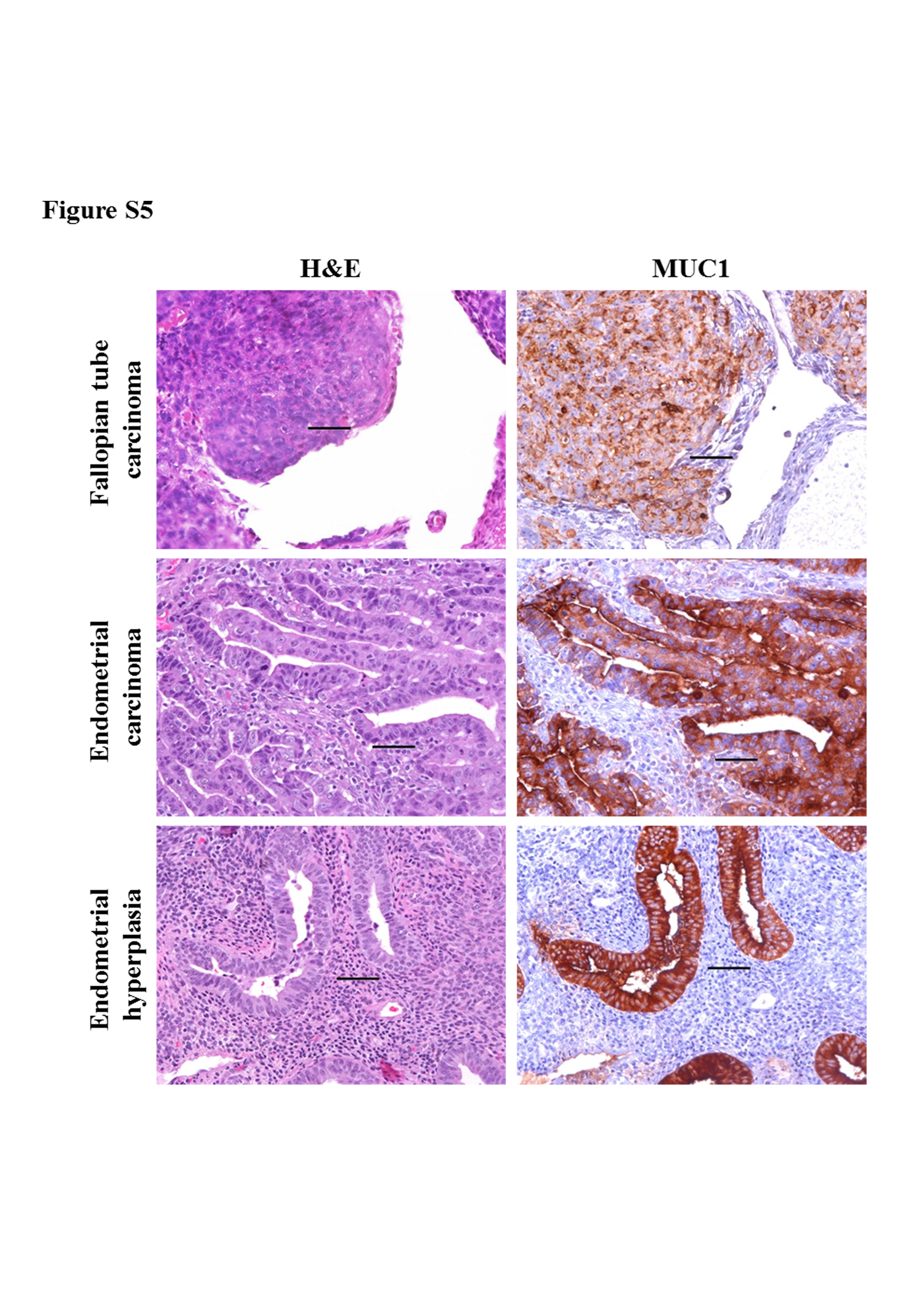

Supplement: Figure S5 — Histomorphology and MUC1 expression in human gynecologic tumors. Left column: HE stains of human fallopian tube carcinoma, endometrial carcinoma and endometrial hyperplasia. Right column: IHC stain for human MUC1 expression. Representative images shown. Scale bar −50 µm. (TIF) [file pone.0102409.s005.tif]
