# Supplementary material for: MUC1 Positive, Kras and Pten Driven Mouse Gynecologic Tumors Replicate Human Tumors and Vary in Survival and Nuclear Grade Based on Anatomical Location
Source: PLoS One. 2014 Jul 31;9(7):e102409. doi: 10.1371/journal.pone.0102409 (PMC4117479; doi:10.1371/journal.pone.0102409)
Supplement: Table S1 — Median survival and number of mice in each tumor group. (DOCX) [file pone.0102409.s006.docx]

**Table S1.** Median survival and number of mice in each tumor group.

| **Tumor site** | **Ovary** | **Oviduct** | **Uterus** |
| --- | --- | --- | --- |
| Median survival, in days | 89 | 82 | 132.5 |
| N  (numbers of animals in each tumor group) | 12 | 9 | 12 |
